# Understanding treatment non-adherence in schizophrenia and bipolar disorder: a survey of what service users do and why

**DOI:** 10.1186/1471-244X-13-153

**Published:** 2013-05-29

**Authors:** Susanne Gibson, Sarah L Brand, Sarah Burt, Zoë V R Boden, Outi Benson

**Affiliations:** 1 SANE mental health charity, 40 Adler Street, London, UK; 2 European Centre for Environment and Human Health, Medical School, University of Exeter, Exeter, UK

**Keywords:** Treatment, Choice, Adherence, Non-adherence, Bipolar, Schizophrenia, Service user, Medication, Mental health

## Abstract

**Background:**

Approximately half of service users with schizophrenia or bipolar disorder do not fully follow treatment recommendations. Studies of adherence have not adequately explored the frequency, consequences and meanings of non-adherence behaviours from service users’ perspectives. This study contributes to a more fine-grained understanding of treatment choices and the support service users require in order to maximise benefit from their medications.

**Methods:**

This was a mixed-methods questionnaire study, employing quantitative and thematic qualitative analyses. Thirty-five individuals with a diagnosis of, and receiving psycho-pharmaceutical treatment for, schizophrenia or bipolar disorder answered online or telephone questions about whether, how, and why they deviated from their treatment recommendations, and what support they currently and would like to receive.

**Results:**

Over half of participants *identified* themselves as being non-adherent, however when asked in detail about intentional and unintentional adherence, 77% reported deviating from treatment recommendations. Critically, 29% were non-adherent *and* satisfied with being so. Service users’ satisfaction with their support was positively correlated with satisfaction with their medication. Participants’ made treatment choices in order to live well. Both side-effects and symptoms could be obstacles to adherence, but feeling well also impacted on participants’ treatment choices. Treatment choices were often made in the context of living well day-to-day, and did not necessarily take into account longer-term effects of non-adherence. Participants wanted more information about their medications, better emotional support (including better access to psychological therapies) and stability in their relationships with health professionals.

**Conclusions:**

This study suggests that non-adherence, both intentional and unintentional, is common amongst individuals with diagnoses of schizophrenia and bipolar disorder, and that this often occurs without health professionals’ knowledge or support. Treatment choices reflect a desire to live well, but are often driven by short-term needs. Given access to more information, and importantly to emotional support, service users could be helped to make treatment choices that adequately reflect the long-term risks of non-adherence, as well as allowing them to live well day-to-day. More research is required better to understand the meanings and complexities of service users’ treatment choices.

## Background

It is well documented that, when it comes to utilising treatments for psychotic illness, service user behaviour does not always coincide with clinical prescription. While there are variations in the way in which adherence is defined and measured, and difficulties in obtaining an accurate measurement [1-3], it is estimated that up to half, and possibly more service users with schizophrenia or bipolar disorder do not follow treatment recommendations[3-6].

Studies of adherence suggest further that there is a correlation between non-adherence and poor outcomes for the patient and his/her social group. Medication non-adherence in particular is thought to raise the risk of psychotic relapse by a factor of three to five [5], and in schizophrenia is associated with rehospitalisation and poor quality of life [4] with the risk of suicide nearly four times higher in service users who are poorly adherent [7]. In bipolar disorder, there is a similar association with relapse, hospital admission and suicide [8,9]. Even allowing for some bi-directionality of the association between non-adherence and poor outcomes [2,3], it is clear that there are good reasons for wanting better to understand and address treatment non-adherence.

While there are a number of specifically targeted interventions aimed at improving adherence, referred to as ‘adherence therapy’ or sometimes ‘compliance therapy’ the UK National Institute for Health and Clinical Excellence (NICE) [10,11] guidelines for the treatment of schizophrenia and bipolar disorder advise *against* using adherence therapy. This may be because of the absence of evidence for their effectiveness: studies of adherence therapy for schizophrenia have shown moderate or no effect on medication adherence, and none on symptom reduction or quality of life [12]. Similarly in bipolar disorder, although recommendations have been made to target knowledge and attitudes about medication and the issue of adherence itself in therapy, Gray et al [13] found that the evidence for the efficacy of such interventions is inconclusive. Berk et al [2] found some evidence of success in psychosocial interventions directly targeting adherence for people diagnosed with bipolar disorder, although they acknowledge that the small number of studies means that there is a lack of a sufficient evidence base. More research has been carried out into interventions where adherence is a secondary outcome. Here the evidence suggests that while some interventions can improve adherence and/or outcomes for people with bipolar disorder, there are a number of variables involved [2].

What service users do is one such variable. Thus rather than treating non-adherence as a conglomerate concept, it is useful to consider the different ways in which service users diverge from treatment recommendations. For example a service user might increase or decrease the amount of medication that they take, and do so either for a short or long period of time. They might change the time at which they take their medication, continue to follow some recommended courses of treatment while not adhering to others, or they might stop taking medication altogether. Adherence behaviour is also something that fluctuates over time [14], and may be intentional or unintentional [2,9]. While these factors impact on the outcomes of non-adherence and success of interventions there is a further, perhaps related dimension to consider. That is, what informs and influences service users’ decision-making and behaviour with regard to adherence and non-adherence?

While understanding both what service users do and how they make and evaluate decisions about following treatment recommendations may be requisite for developing and targeting interventions that are successful in improving adherence [2,9], added to this is a concern to ensure that treatment decisions are based on a collaborative therapeutic alliance that takes into account the perspective of the service user. Thus it is noted that there has been a move away from the language of ‘compliance’ to that of ‘adherence’, reflecting the role of the service user within the therapeutic relationship in discussing and agreeing a course of treatment, and in deciding to follow the recommendations [2,15].

Taking this further, the recovery model looks beyond treating symptoms and preventing relapse in severe and enduring mental illness to a more holistic view that includes establishing or re-establishing an integrated sense of self as competent and self-directing [16,17]. Here the need for a collaborative approach to treatment based on an understanding of the first-hand experience of the service user is twofold: first in determining what recovery means to that person, and second in facilitating a sense of agency.

This paper reports on a study investigating first person accounts of treatment adherence decisions and behaviours among service users with a diagnosis of schizophrenia or bipolar disorder, and their perspectives on available and desired support to maximise benefit from their treatment. By eliciting some of the more fine-grained aspects of service users’ treatment choices, the study contributes to an approach that takes seriously the role of the service user in successfully managing and living with a severe and enduring mental illness.

## Method

### Participants

Forty-one people living in England with both a diagnosis of and receiving treatment for either schizophrenia or bipolar disorder were recruited. Thirty-five reported receiving psycho-pharmaceutical treatment and their responses are reported in this paper (N = 35). Participants were recruited via the SANE website, and through publicity in SANE communications, including social media.

The majority of participants defined themselves as White British (n = 23), with 1 reporting their ethnicity as White English, 1 as Asian Indian, 1 as Asian Pakistani, 1 as Welsh, and eight did not respond. Ten reported having a diagnosis of schizophrenia and 24 a diagnosis of bipolar disorder. One participant reported having a diagnosis of both schizophrenia and bipolar disorder. Twenty-five were being treated by a psychiatrist, 17 were being treated by a primary care physician, 1 was being treated as an inpatient, and 14 as outpatients. Four were also receiving individual therapy, 1was in group therapy, and 1 was receiving both.

### Ethics

The study received ethical approval from the North London Research Ethics Committee 2 (REC reference number: 10/H0724/37). Participants gave their informed consent online by confirming (by clicking in the appropriate box) that they had read and understood the Participant Information Sheet. It was not possible for participants to progress with the online study until they had given their consent. Participants taking part by telephone completed the same procedure verbally with the researcher, having been sent a copy of the Participant Information Sheet in advance. Participants were informed that their responses may be published, but their anonymity would be protected.

### Design and measures

This was a mixed-methods questionnaire study. The questionnaire was delivered online or via telephone, and aimed to gather information about how often, in what way, and why service users deviate from their treatment recommendations. Up to 42 multiple choice and open-ended questions asked service users to give their own reasons related to a variety of prescribed possible ways of regulating their medication [see Additional file 1]. The questionnaire asked participants about both psycho-pharmaceutical and non-medical treatments; the results that we report on here are derived from the questions concerning the former. Intentional non-adherence and unintentional non-adherence were both explored, and service users were also asked about their expectations of what would happen, and what actually happened when they deviated from treatment recommendations. Participants were also asked about the extent to which they discuss their treatment non-adherence with their health care professional, and what informed those decisions. Finally, they were asked about available and desired support in relation to their diagnoses.

Adherence was measured by self-report [14]. Service users were asked whether they followed treatment recommendations exactly, or did something different. They were also asked to describe their intentional and/or unintentional non-adherence and to estimate the frequency of each.

### Data analysis

Quantitative data was explored primarily with descriptive statistics. Chi-square analyses and non-parametric correlations were undertaken where feasible and useful (all with a threshold of p < 0.05).

Qualitative data was explored using a thematic analysis following the guidelines of Braun & Clarke [18]. Data was initially coded inductively, before codes were clustered into themes and subthemes. Themes were developed on the basis of their prevalence across the data-set, and with reference to potential theoretical interests. Qualitative analysis was initially carried out by one author, then, to increase validity, themes were independently checked against the data by the other researchers. Any differences of opinion were discussed and resolved by all authors. There were no instances of disagreement on themes which were not resolved satisfactorily. Participant quotations were chosen to best illustrate the theme under discussion.

## Results

### Quantitative

Service users were asked how closely they followed their treatment recommendations, and 46% (n = 16) reported following recommendations exactly. However, when asked to describe the different ways in which they did something different to recommendations, while 54% (n = 19) reported intentional non-adherence, 71% (n = 25) reported unintentional non-adherence, with a total of 77% (n = 27) doing something different to recommendations. That is, half of those (n = 8) who reported following treatment recommendations exactly then went on to describe occasions on which they were unintentionally non-adherent. Of those who reported intentional non-adherence, roughly half were intentionally non-adherent at least twice a month; likewise, of those who were unintentionally non-adherent, roughly half were unintentionally non-adherent at least twice a month (Table 1).

**Table 1:** What service users do: self-reported service user behaviours

**Self-reported service user behaviour**	**Service users**
	**(no./total (%))**
Service users who reported following recommendations exactly	16/35 (46)
Service users who described intentionally and/or unintentionally doing something different to recommendations	27/35 (77)
Service users who described intentionally doing something different to recommendations	19/35 (54)
Of these	
• 2 or more times a month	9/19 (47)
• Intentionally taking less medication	12/19 (63)
• Intentionally taking more medication	12/19 (63)
Service users who described unintentionally doing something contrary to recommendations	25/35 (71)
Of these	
• 2 or more times a month	12/25 (48)
• Forgot to take medication	17/25 (68)
• Symptoms prevented taking medication	12/25 (48)
• Unable to motivate self to take medication	12/25 (48)
• Practical reasons	9/25 (36)
• Unsure about recommendations	5/25 (20)
• Lost prescription	1/25 (4)

Service users reported their expectations and the outcomes of intentionally doing something different to treatment recommendations. On 55% of those occasions when service users had a positive expectation (including expectations of no change), they also experienced a positive outcome (including outcomes of no change). On 45% of those occasions when service users expected a positive outcome (including expectations of no change), they experienced a negative outcome. On all of those occasions when service users had a negative expectation, they then experienced a negative outcome. Notably, while on the majority of occasions of intentional non-adherence, service users had positive expectations, there were still some occasions (n = 5) on which service users chose not to follow recommendations despite expecting negative consequences (Table 2).

**Table 2:** Service user expectations and outcomes: self-reported service user behaviours

**Self-reported service user behaviour**	**Occasions of intentional non-adherence (no. / total (%))**
Occasions on which service users’ expectation of a positive outcome was followed by an outcome of the same valence	18/33 (55)
Occasions on which service users’ expectation of a negative outcome was followed by an outcome of the same valence	5/5 (100)
Occasions on which service users’ expectation of a positive outcome was followed by an outcome of negative valence	15/33 (45)
Occasions on which service users correctly predicted positive or negative valence of the outcome of doing something contrary to recommendations.	23/38 (61)

Thirty-four percent (n = 12) of service users reported that they would like to change the way they followed their treatment recommendations, with 20% (n = 7) wanting to follow them more closely and 14% (n = 5) less closely. Sixty-six percent (n = 23) did not want to change the way they followed recommendations. Of those who did not want to change the way they followed their recommendations, only 57% (n = 13) reported that they followed recommendations exactly. This means that nearly a third of service users (29%, n = 10) did not take medication as recommended *and* were happy with their level of adherence (Table 3).

**Table 3:** **Service user desire to change**/**not change treatment behaviour**

**Service user self-report**	**Service users**
	(no./total (%))
Did want to change the way they followed recommendations	12/35 (34)
• Follow more closely	7/35 (20)
• Follow less closely	5/35 (14)
Did not want to change the way they followed recommendations	23/35 (66)
Of these	
• Doing something different to recommendations	10/23 (43)
Not following recommendations *and* did not want to change their level of adherence	10/35 (29)

**Figure 1:**
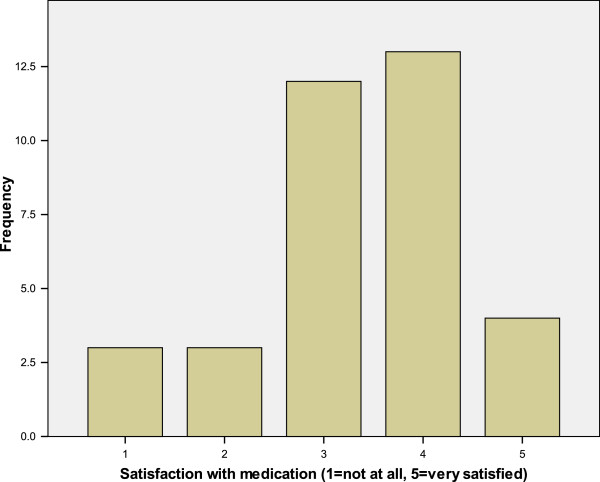
Service user satisfaction with medication.

**Figure 2:**
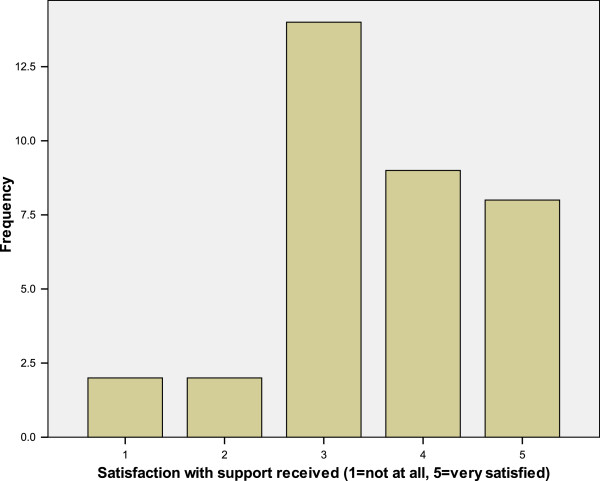
Service user satisfaction with support.

Service users were asked to rate their satisfaction with their current medication on a scale of 1 (not at all satisfied) to 5 (very satisfied) (Figure 1). They were also asked to rate their satisfaction with their current support (Figure 2). There was a significant positive correlation between satisfaction with support and satisfaction with medication received (r_S_ = 0.43, N = 35, p = 0.01).

### Qualitative: Thematic analysis

Three main themes were identified: ‘Living well for self and others’; ‘Obstacles to adherence’; ‘Therapeutic support’.

#### Living well for self and others

Subthemes: ‘Staying well and avoiding negative consequences’; ‘Managing side-effects and symptoms’

The reasons service users gave for either following or intentionally not following treatment recommendations can be analysed under the theme ‘Living well for self and others’. That is, service-users made decisions about their treatment recommendations in order to live as well as possible with the symptoms of their diagnosis and the side-effects of medications, with decision-making taking place in response to the demands of everyday living.

The reasons given by service users for always or sometimes following recommendations formed the subtheme ‘Staying well and avoiding negative consequences’. Treatment recommendations were adhered to insofar as medication was seen as necessary to stay well and live as good a life as possible or insofar as it was necessary to avoid negative consequences

“To stay in one piece, stay balanced” (P13)

“In order to get the very best out of life and remain safe” (P1)

“Meds keep my head just above water, failing taking them…I and people around me pay the price” (P41)

“Scared of being sectioned and I dislike some of the Schizophrenia symptoms” (P35)

This subtheme was further supported by service users’ reasons for satisfaction with their current medication

“As long as I’m on medication I haven’t been ill” (P11)

“I started to get my life back and have some degree of normality” (P39)

Conversely, dissatisfaction with current medication appeared to reflect the ways in which medication failed to support ‘living well’, for example problems with side-effects, lack of efficacy in controlling symptoms or the burdens associated with following a treatment course:

“long term health effects and side effects. It’s toxic” (P28)

“I was once very active and went to the gym 4 times a week, now I have become lethargic and fatigued from my medication” (P23)

“I still suffer from intrusive and painful thought disturbance” (P1)

“… monthly or fortnightly my mood drops and I have a severe depression lasting about a week” (P41)

“I am really fed up with … having to have a blood test every four weeks” (P35)

*“I just wish I didn’t need to take so much medication*” *(P19)*

The reasons given by service users for intentionally doing something different to their treatment recommendations informed the subtheme ‘Managing side-effects and symptoms’. Here there was still a concern with living well, and changes were made in order to manage side-effects and symptoms in a way that enabled service users to live with the difficulties associated with their diagnosis and medication. Those who took more medication tended to frame their reasons in terms of managing symptoms; those who took less mediation tended to frame their reasons in terms of managing side-effects, although some service users reported taking less medication because they felt well

“I started feeling depressed and wanted to increase my dose so I slept through the day” (P18)

“Intensity of voices made it hard to cope” (P3)

“Didn’t like the sedative side effects” (P15)

“I had a meeting at work the next day so skipped my evening dose” (P9)

“I had been coping well for a significant period of time” (P33)

“I feel okay on less and see no reason to take more” (P39)

Changing the time of medication tended to be associated with managing sleep patterns and tiredness

“I was working nights and needed to feel alert during the night” (P9)

“Took antidepressants once a day rather than twice a day, to have more energy during the day” (P3)

#### Obstacles to adherence

Subthemes: ‘Feeling well enough’; ‘Contending with side-effects and symptoms’

Service users were asked to select from a list of options that best described the reasons for their unintentional non-adherence (Table 1). Analysis of the service users’ accounts of their unintentional non-adherence suggested the theme ‘Obstacles to adherence’ with 2 subthemes, ‘Feeling well enough’ and ‘Contending with side-effects and symptoms’.

Thus although 17 service users reported that they had forgotten to take medication (Table 1), within this group the experiences described differed notably. For some, forgetting appeared to be a function of ‘Feeling well enough’, that is, of a remission of symptoms and/or being busy or generally engaged with everyday life

“Just forgot too busy at work” (P15)

“Somehow I forgot to take the medication, maybe because I was feeling well” (P25)

For others ‘forgetting’ was part of the experience of “Contending with side-effects and symptoms”

“last night I forgot to take my lithium because I was too tired and didn’t want to feel sick” (P23)

“Last week, was awake for three days did not take any medicine during this period” (P14)

This also incorporated aspects of loss of motivation:as well as the experience of symptoms directly impacting on adherence, including delusional thoughts or fears about medication, hopelessness, hallucinations, mania or beliefs about the impact of non-adherence on symptoms

“too mentally and physically tired to get out of bed and fetch medication/water” (P27)

“I didn't feel like doing anything and taking medication was one of those things” (P37)

“I wanted to know what I really thought and I believed that the medication was controlling my thoughts - so it had to be stopped” (P35)

“Too low felt there was no point as was going to kill myself anyway” (P15)

“I am hearing bad voices and they sometimes tell me that the medication is poison” (P7)

“I was high as a kite” (P4)

“Thought I might feel better if dose missed occasionally” (P16)

Further, where service users reported practical difficulties these might sometimes be understood as combining with side-effects or symptoms with a resulting increased impact

“I was stuck in a flat with little energy after being on two lots of medication sleeping 16 hours or more with no transport” (P24)

One shared element of the two themes presented so far is the way in which decision-making and behaviour tends to be in response to the day-to-day challenges and demands of living with a severe and enduring mental illness, rather than on longer term considerations. This will be discussed below.

#### Therapeutic support

Subthemes: ‘Enabling and disabling communication’; ‘Supporting the person’

The role of the therapeutic relationship featured in service user accounts in a number of ways, analysed under the theme ‘Therapeutic support’ with the subthemes ‘Enabling and disabling communication’ and ‘Supporting the person’.

Good experiences of communication were enabling in the sense that the service users felt supported and better able to manage their illness. However, in other cases communication was experienced as impaired and impairing, such that service users felt unable to communicate, or where attempts to communicate were met with a less than satisfactory response. Also informing this was the desire for more and better information to help support treatment choices.

Hence service users were asked whether they discussed the times when they had not followed treatment recommendations with their doctor. Some of the reasons for not discussing non-adherence related to concerns about the consequences, accessibility of health care professionals, or to the service user’s own state of mind

“in fear he may take me off the haloperidol” (P7)

“frightened of being sectioned” (P27)

“I don’t see the doctor for another 6 months” (P3)

“I didn’t want to interact with anyone” (P18)

“too ashamed” (P26)

In other cases service users described a relationship in which they felt trusted to make their own decisions, or appeared to trust themselves to make the decision

“When the ground rules were established with my recent GP, it was discussed that I could increase or decrease (by a small amount) my medication as and when required” (P33)

“the medication dosage works and I see no reason to change as I am not taking too much” (P8)

Where service users had discussed their non-adherence, this had in a number of cases resulted in a positive outcome

“my psychiatrist is going to review my anti d[epressant]” (P7)

“Slow release anti-depressant prescribed. So I only need to take it once a day” (P3)

However, in other cases there were blocks to communication, or communication resulted in a negative outcome

“I told the Dr the symptoms but wasn't honest about what medication I was taking less of” (P18)

“They were not willing to listen to the prescription mess up that had occurred and just said I had decided to stop taking meds myself” (P37)

“I was ‘bullied’ into taking a different medication” (P35)

The desire for more and better information to help support treatment choices included wanting to be informed about alternative treatments, more information about the long term effects of medication, and information about the effects of taking medication while pregnant

“Independent, unbiased information about my medication and other possible treatments” (P3)

“Information about long term consequences of the medications” (P10)

“Access to studies about my medication and pregnancy” (P9)

However, although making informed choices was perceived as important and providing information part of the role of the healthcare professional, there was also a clear need for emotional support, including stability of care, which informed the subtheme ‘Supporting the person’.

There were a substantial number of positive experiences of support from healthcare providers

“My psychiatrist is fantastic with email access and I have her mobile number” (P15)

“I have had excellent support from my GP and know that I could call him anytime and he would help in any way he could” (P33)

“I have access to different types of support when I need it and also touch base with professionals to check all is ok. I know I can ring my care co-ordinator whenever I need to” (P37)

Others described mixed experiences or a need for improved therapeutic support

“CPN very supportive. Psychiatrist being changed all the time” (P14)

“I get good support from my psychiatrist and my CPN but my GP is not approachable or involved” (P35)

“would like a CPN again (I used to have one) or a support worker/someone to talk to” (P27)

“I am not listened to regarding the side effects of my medication and no peer group support has been offered” (P9)

“I don't get any support unless I ask for it, and sometimes I am so cross I don't feel I can in what is considered an appropriate way, The doctor does not know me well, I had to change due to moving and they don't try to understand” (P2)

Here, being listened to and understood appears to be an important part of feeling supported, and a number of service users pointed to the lack of talking therapies. In addition, when asked what additional support they would like, participants expressed a desire for more talking therapies

“Do have some support but would prefer regular counselling and transport to it” (P24)

“I get medical attention I think I need except talking therapies” (P13)

“I think being offered some type of group or individual therapy would be really beneficial” (P18)

“More talking therapy and counselling to give me peace of mind” (P1)

## Discussion

### What do service users do and why?

Previous studies suggest that around 50% of service users are non-adherent, although as it was noted above, there are competing definitions and different measurements of adherence, as well as difficulties in obtaining an accurate measurement [3,5]. This study reflects these findings, with 46% of service users reporting that they follow treatment recommendations exactly. However, of these, half then described occasions on which they had unintentionally done something different to recommendations. That is, in this study, service users reported that they followed treatment recommendations exactly unless they considered that they were *intentionally* doing something different to their recommendations. Of those who had intentionally done something different to recommendations, roughly half reported doing so twice a month or more. Of those who had unintentionally done something different, roughly one third reported doing so twice a month or more (Table 1).

The accounts that service users gave of their reasons for adhering and deliberately not adhering to treatment recommendations informed the theme ‘Living well for self and others’. Again in line with previous studies, the reasons given for following recommendations were perceived efficacy of medication in controlling symptoms and enabling wellness, and a desire to avoid negative outcomes, including relapse and negative social consequences [5,9,15]. This was further reflected in the reasons given for satisfaction with medication.

Service users’ accounts of their reasons for intentionally doing something different to recommendations informed the subtheme ‘Managing side-effects and symptoms’, while in cases of unintentional non-adherence, some service users appeared to be ‘Contending with side-effects and symptoms’. Participants described the impact of psychotic, manic and depressive symptoms on adherence. While non-adherence is known to be correlated with the manic phase in bipolar disorder, there is a lack of understanding of the relationship between depressive symptoms and non-adherence [2]. In this study depressive symptoms were given as reasons both for taking more and taking less medication, and for intentional and unintentional non-adherence.

That the same symptoms can impact on both intentional and unintentional non-adherence might support Basco & Smith’s [14] claim that there is not always a clear distinction between intentional and unintentional non-adherence. However, it appears that there is an important difference in the way in which symptoms are experienced as reasons for non-adherence, which we have analysed by contrasting ‘managing symptoms’ with ‘contending with symptoms’. Thus for example, for one service user depressive symptoms informed the decision to take more medication when they were *“feeling very low and recognize[d] the signs of spiraling into a depression” (P33)*, while for another it prevented their following recommendations as they “*didn't feel like doing anything and taking medication was one of those things” (P37)*. Likewise, one service use took more medication because *“intensity of voices made it hard to cope” (P3)* while for another *“voices instructed me not to take the tablets” (P14).* This points to the complexity of service users’ lived experience of managing their medication and the symptoms of their illness.

An absence of symptoms also impacted on whether service users’ followed treatment recommendations. That is, in some cases, ‘feeling well enough’ appeared to present an obstacle to adherence. The relevance for adherence of accepting a diagnosis and coming to terms with the implications of managing a long-term severe and enduring mental illness has been recognised [15]. One participant’s description of having forgotten to take their medication seems to acknowledge explicitly that this is a factor in determining treatment behaviour, saying *“I missed a few days because (a) I was extremely busy and distracted and (b) subconsciously I didn't want to take it because I occasionally become very resentful of the fact that I have to take medication every day” (P33)*.

In their review, Clatworthy et al [9] found that concerns about side-effects were associated with non-adherence in bipolar disorder and DiBoventura et al [19] found a significant association between self-reported side effects and non-adherence in people with schizophrenia, in particular extra pyramidal symptoms and agitation, and metabolic side effects such as weight gain. In this study intentional non-adherence was associated with a wish to avoid side effects such as tiredness and feeling sedated; physical side effects such as weight gain and agitation were among the main reasons given by those participants who reported a low level of satisfaction with their medication.

One question is whether the impact of side-effects on service users’ treatment choices represents a reasoned weighing of relevant considerations or whether it should instead be construed as an absence of adequately informed decision-making [3,14]. Some appear to take it as evidence of the latter. For example, Pope & Scott [6] distinguish between side-effects and fear of side-effects informing decision-making and Basco & Smith [14] suggest that a memory of unpleasant side-effects might inform an affective rather than reasoned response. In this study, where participants described intentional non-adherence, they appeared to refer to decision-making informed by current rather than remembered or conjectured side-effects suggesting that the undoubtedly real side-effects informed rather than skewed service users’ decisions. This is discussed further below in the context of the time-scale for service user decision-making.

First, however, we consider further the context for service user behaviour and decision-making.

### Understanding service user treatment choices

The idea of a collaborative approach to treating severe and enduring mental illness, and the concept of recovery, point to a need to understand not only the nature of and reasons for treatment adherence and non-adherence, but also the broader context in which service users’ decision-making and behaviour takes place.

Critically, this study found that nearly one-third of service users with bipolar disorder or schizophrenia were *both* non-adherent and satisfied with being so. Thus while the literature points to the negative impact of non-adherence, at least some participants appeared to have found a way of adjusting their treatment that they didn’t want to change. While it is clear that there is an association between non-adherence and poor outcomes, this aspect of service user experience requires further investigation. For example, it has been pointed out that there may be a bi-directional relationship between non-adherence and relapse with the possibility that adherence decreases when the service user is becoming unwell, as well as non-adherence leading to relapse in some cases [2]. If it is the case that the causal relationship between non-adherence and poor outcomes is not straightforward, then one explanation for service user satisfaction with non-adherence might be that, on some occasions at least, non-adherence does not result in a poor outcome.

Alternatively, this aspect of service user experience might be explained as a function of another finding from this study. That is, that service user treatment choices occur on a daily basis in response to the everyday demands of ordinary living, for example taking less medication in order to stay alert, or taking more in order to sleep through depressive feelings. That is, it appeared that decisions not to follow treatment recommendations were made in order to live well by balancing side effects and symptoms on a day-by-day basis, rather than to support long-term goals such as preventing relapse.

Further, where it was found that service users’ choices about medication self-regulation were in most although not all cases informed by realistic expectations about the positive or negative valence of the outcome, expectations and outcomes of non-adherence were again focused on the short-term rather than the long-term. Thus while this suggests that many service users self-regulated their medication in response to a relatively realistic weighing up of the short-term, or day-to-day costs and/or benefits of non-adherence, it leaves open the question of whether these were taken to outweigh longer-term considerations such as the risk of relapse, or whether longer term considerations were not taken into account (although it is worth noting that where participants expressed a desire for more and better information, this included information about the long-term effects of taking medication).

Again, the focus on day-to-day decision-making can be analysed in different ways. One possibility is to interpret service users’ reasoning as flawed, insofar as the longer term risks of non-adherence are not given sufficient weight against the expected short term benefits. Another, perhaps more appropriate understanding attends to the significance of the day-to-day experience of managing a severe and enduring mental illness, where balancing side-effects and symptoms in order to respond to the demands of everyday living is as important as longer term goals such as preventing relapse. Thus Basco & Smith [14] suggest that understanding non-adherence requires attention to the daily obstacles and decision-making that informs behaviour; extending this point further it might be argued that understanding what recovery means for a service user requires an understanding of the everyday experiences that are the building blocks of a life well lived. This is not to say that encouraging service users to take long-term as well as short-term outcomes into account is misguided, but rather that what is required is an expanded perspective on the context for service user treatment choices in order to support those choices and the desire to live well.

### Supporting service user treatment choices

One implication of the daily focus of service users’ treatment choices is a conflict of time frame between service user decision-making and access to clinical support and expertise. There are of course practical limits to the availability of direct support from health care professionals, and where the need for ‘Therapeutic support’ consists in a desire for more and better information, there are a number of ways in which this might be provided, for example through an online resource such as the ‘Choice and Medication’ website (http://www.choiceandmedication.org/cms/). However, it is clear that the need for support from health care professionals extends beyond providing advice and information to ‘Supporting the person’

The therapeutic relationship has been seen by some as an important predictor of adherence [2,15]. However, this study suggests that while the therapeutic relationship is important to service users, the quality of the alliance is a factor in their satisfaction with support (which is in turn correlated with satisfaction with medication), rather than for treatment adherence as such. This can be compared with a recent study of the correlation between clinician and patient ratings of the therapeutic relationship and treatment adherence in schizophrenia [4]. While both clinician and patient ratings of the therapeutic relationship were linked with adherence (i.e. a higher rating correlated with better adherence), there was a stronger correlation between clinician ratings and adherence than between patient ratings and adherence. As the authors of the report suggest, one explanation for this is that clinicians might take a more positive view of the therapeutic relationship in part because a patient is adherent; the patient on the other hand might well base their assessment on broader criteria.

In the light of this, it is also interesting to note that while fewer than 20% of service users in our study received non-medical treatment, this was highlighted as one of desired areas of additional support. The UK All Party Parliamentary Group on Mental Health [20] recently identified lack of access to psychological therapies for people with schizophrenia as one obstacle to implementing NICE guidelines, and even if the evidence supporting the relationship between psychological therapies and *adherence* is equivocal, there is evidence that psychotherapy can improve overall outcomes for people with bipolar disorder and schizophrenia [3,16]. Further, Lysaker & Roe [21] argue for the role of psychotherapy in a broader concept of recovery that looks beyond the control of symptoms to “the recapture of a coherent personal narrative and/or of metacognitive capacity”. We suggest that psychotherapy that focuses on building a coherent sense of self as a person with both future goals and immediate needs would also aid in helping service users to integrate short-term and long-term decision-making with regard to their treatment recommendations.

#### Limitations

Limited resources meant that a relatively small number of participants were recruited to the study, such that statistical analyses lacked power. For example, significant associations between service users’ adherence and items such as satisfaction with medication and satisfaction with support were not found. Likewise no significant associations were found between service users’ diagnoses and reasons for non-adherence. A larger study would allow further statistical analysis and further qualitative exploration of the experiences of people with different diagnoses and different levels and types of adherence and non-adherence.

As this study focused on service users’ perspectives and the ways in which they described and understood their own behaviour, adherence was measured by self-report; that is participants were asked whether they followed treatment recommendations exactly, and if they did something different, to estimate how often. Use of a validated adherence measure might have given a more accurate result. However, since the aim was not primarily to measure adherence but to understand the context for service user treatment choices, it was considered that the disadvantages of asking participants to complete an adherence measure, particularly with regard to encouraging them to complete the questionnaire, would outweigh the advantages.

## Conclusion

Whilst medication non-adherence carries serious risks for service users, more than half of service users taking medication for either schizophrenia or bipolar disorder do something different to their treatment recommendations.

This study suggests that the reality for people with a diagnosis of schizophrenia or bipolar disorder is that managing their illness and living well requires balancing side effects and symptoms, and that this in many cases means at least occasionally departing from treatment recommendations. Where service users were intentionally and/or unintentionally non-adherent, this was usually in response to the day-by-day challenges of ordinary living, standing in stark contrast to the time frame according to which clinical interactions tend to take place. While many service users reported good experiences of clinical support, there was a perceived need for more extensive provision in this respect, including greater access to talking therapies.

Future research should focus on developing interventions to help service users to understand and manage the risks, and to offer them non-judgemental information, support and advice.

## Competing interests

There are no competing interests.

## Authors’ contributions

OB conceived of and contributed to the design of the study. SLB and SG jointly designed and constructed the questionnaires. SB and SLB carried out the data analysis, with additional analysis undertaken by SG and ZB. SLB and SG wrote the final manuscript, with contributions from OB and ZB. All authors read and approved the final manuscript.

## Pre-publication history

The pre-publication history for this paper can be accessed here:

http://www.biomedcentral.com/1471-244X/13/153/prepub

## Supplementary Material

Additional file 1 Understanding service user treatment choices service user survey. Click here for file
