# Supplementary material for: Understanding treatment non-adherence in schizophrenia and bipolar disorder: a survey of what service users do and why
Source: BMC Psychiatry. 2013 May 29;13:153. doi: 10.1186/1471-244X-13-153 (PMC3695802; doi:10.1186/1471-244X-13-153)
Supplement: Additional file 1 — Understanding service user treatment choices service user survey. [file 1471-244X-13-153-S1.pdf]

Additional File 1

UNDERSTANDING SERVICE USER TREATMENT CHOICES SERVICE USER SURVEY

Service User Questionnaire Web Page Structure

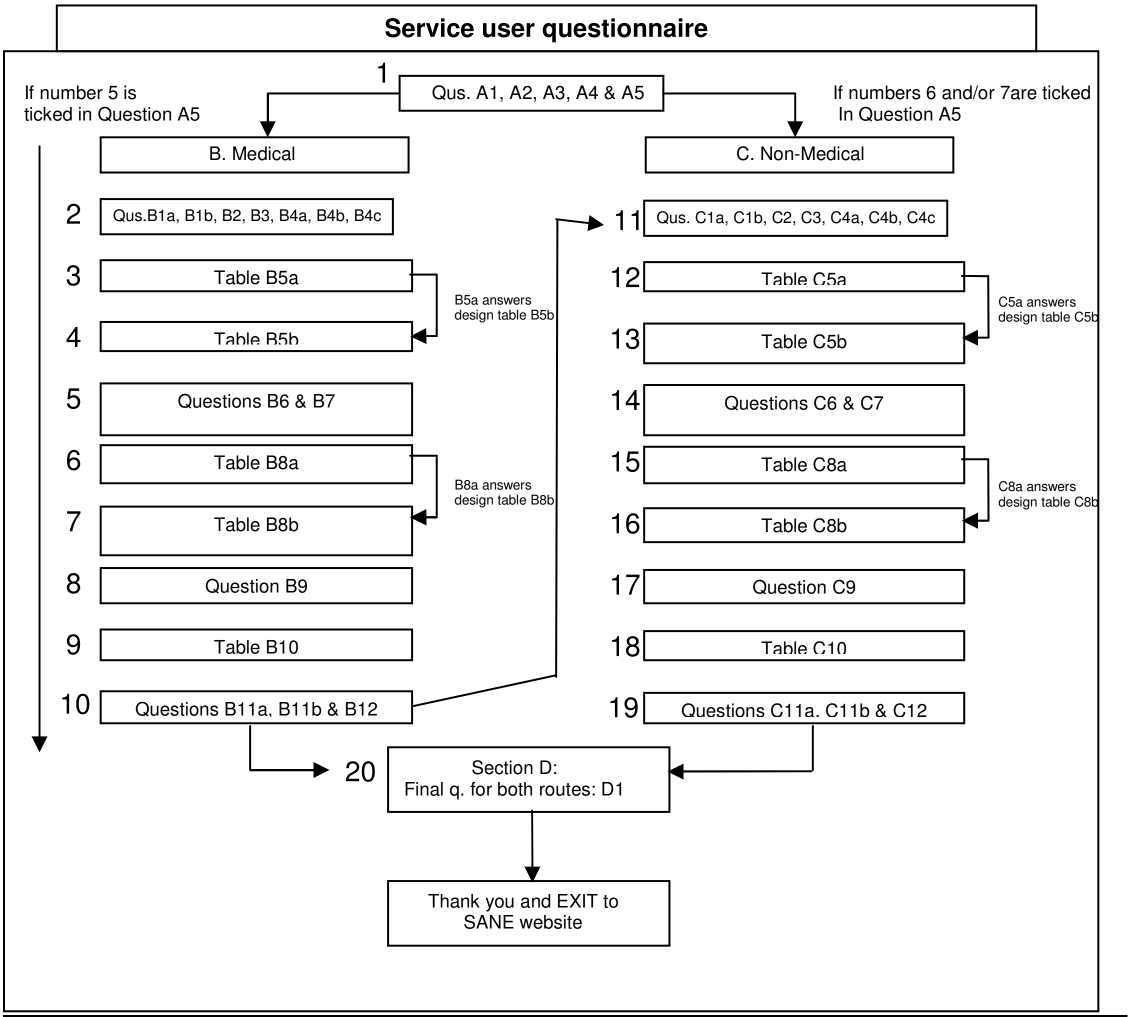

EXPLANATORY NOTES

Each numbered box corresponds to one webpage

The questionnaire is divided into sections A, B, C and D. All participants will be asked to complete sections A and D. Depending on the answers given to question A.5., participants will be asked to complete section A, or section B, or both (i.e. depending on whether they are receiving only medical treatment, only non-medical treatment, or a combination of the two).

Tables B5b, B8b, C5b and C8b will be automatically generated depending on the selections made for tables B5a, B8a, C5a and C8a respectively. The number of rows appearing in tables B5b, B8b, C5b and C8b will correspond with the number of selections made in tables B5a, B8a, C5a and C8a.

[service user questionnaire webpage 1]
SECTION A

A.1. How long have you been diagnosed with this condition?

☐ Less than 1 year
☐ 1-5 years
☐ 6-10 years
☐ More than 10 years
☐ I don't know

A.2. What is your gender?

☐ Male
☐ Female

A.3. Who, if anybody, do you currently live with?

☐ Partner/spouse
☐ Family members(s)
☐ Friends
☐ I live on my own
☐ Other (please specify): [ ]

A.4. How would you describe your ethnic background?

☐ White British
☐ White English
☐ White Welsh
☐ White Scottish
☐ White Irish
☐ White European
☐ Any other White (specify below)
☐ Asian British
☐ Asian Indian
☐ Asian Pakistani
☐ Asian Bangladeshi
☐ Any other Asian (specify below)
☐ Black British
☐ Black Caribbean
☐ Black African
☐ Any other Black (specify below)
☐ Chinese
☐ Any other ethnic group (specify below)
☐ Mixed, white and Black Caribbean
☐ Mixed, White and Black African
☐ Mixed, White and Asian
☐ Any other mixed background (specify below)
☐ I don't know

Specific ethnic background: [ ]

A.5. What types of treatment or services do you currently receive? [Select all that apply]

☐ I am currently in a psychiatric hospital receiving inpatient care
☐ I am seen regularly by a psychiatrist
☐ I am seen regularly by a primary care physician (GP or similar)
☐ I have regular appointments at a mental health clinic, day centre or outpatient facility
☐ I am prescribed medication for schizophrenia and/or bipolar disorder

- ☐ I receive some form of talking therapy from a qualified counselor or a psychologist
- ☐ I participate in group therapy sessions
- ☐ Other. Please tell us more or tell us about any other services you are accessing, or treatments you are receiving:

[service user questionnaire webpage 2]
SECTION B

*******Section B only appears if the fifth box in question A.5. is ticked*******

Experience of treatment I: Pharmaceutical treatment.
Please answer the questions in the following section in relation to medication (e.g. pills and/or injections) which you are prescribed for Bipolar Disorder or Schizophrenia.

B.1a. Generally speaking, how satisfied are you with your current medication? Please put a number between 1 and 5 in the box below, with 1 being not at all satisfied, and 5 being very satisfied.

☐

B.1b. Please tell us briefly the **main** reasons why you are satisfied and/or not satisfied with your current medication/s.

☐

B.2. When your medication was prescribed did your doctor discuss your treatment choices with you?

Yes/No

If yes, how involved did you feel in the decision about your medication? Please put a number between 1 and 5 in the box below, with 1 being not at all involved, and 5 being very involved.

☐

B.3. Generally speaking, when you are prescribed medications, how closely would you say you follow the recommendations of your doctor?

☐ I follow the recommendations **exactly**
☐ I **mostly** take my medicines as recommended
☐ I do something that is **different** to the recommendations **quite often**
☐ I do something that is **different** to the recommendations **most of the time**
☐ I **never** follow the recommendations

B.4a. Please tick the statement that best describes how you feel about the way in which you follow the recommendations of your doctor.

☐ I would like to follow my medication recommendations **more** closely than I do
☐ I am satisfied with the way I follow my medication recommendations and **do not want to change** how closely I follow them
☐ I would like to follow my medication recommendations **less** closely than I do

B.4b. [If answer to 12a is first or third box:] What help would you need to make the change you want to make?

B.4c. [If answer to 12a is first or third box:] What kind of things have prevented you from following your treatment recommendations in the way that you would like to?

[service user questionnaire webpage 3]

B.5a. Below are some options for ways in which you might **deliberately** take your medicines in a way that is different to what has been recommended to you by your prescribing doctor. Please tick any that have at some time applied to you.

Options (tick box for all that apply)	
**None Apply** - I have never deliberately avoided taking my medication.	☐
**None Apply** - I **always** follow the treatment course I am prescribed	☐
I took **less** medication than prescribed for a **short** period of time (i.e. less than a week)	☐
I took **less** medication than prescribed for a **long** period of time (i.e. a week or more)	☐
I took **more** medication than prescribed for a **short** period of time (i.e. less than a week)	☐
I took **more** medication than prescribed for a **long** period of time (i.e. a week or more)	☐
I changed the time of day I took my medication	☐
I stopped my medication earlier than recommended	☐
I **Never** follow the treatment course I am prescribed	☐
I **Changed** my behaviour from one pattern to another, e.g. I used to take more than recommended but stopped doing that	☐

[service user questionnaire webpage 4]

Only those options ticked in the previous question appear in the table here.

B.5b Below are the options you ticked for ways in which you might **deliberately** take your medicines in a way that is different to what has been recommended to you by your prescribing doctor. Please describe the most recent time you did this. What were your reasons, what did you expect to happen and what did actually happen? Please also indicate whether you discussed the change with your prescribing doctor and if you did, what the result of the discussion was. If you did not discuss the change with your doctor, please tell us why not. For each option, if you can, please tell us which phase of your illness you were in at that time.

Options (tick box for all that apply)	*Description of most recent time, including your reasons*	*Expectations*	*What actually happened as a result*	*Discussed with doctor? Outcome of discussion or reasons for not discussing*	*Phase of illness Bipolar Disorder (Please tick)*	*Phase of illness Schizophrenia (Please tick)*
I took **less** medication than prescribed for a **short** period of time (i.e. less than a week)				□ **Yes**<br>How did the discussion go?<br>□ **No**<br>Reasons why not	□ Manic<br>□ Depressive<br>□ Mixed<br>□ Latent	□ Acute<br>□ Stable
I took **less** medication than prescribed for a **long** period of time (i.e. a week or more)				□ **Yes**<br>How did the discussion go?<br>□ **No**<br>Reasons why not	□ Manic<br>□ Depressive<br>□ Mixed<br>□ Latent	□ Acute<br>□ Stable
I took **more** medication than prescribed for a **short** period of time (i.e. less than a week)				□ **Yes**<br>How did the discussion go?<br>□ **No**<br>Reasons why not	□ Manic<br>□ Depressive<br>□ Mixed<br>□ Latent	□ Acute<br>□ Stable
I took **more** medication than prescribed for a **long** period of time (i.e. a week or more)				□ **Yes**<br>How did the discussion go?<br>□ **No**<br>Reasons why not	□ Manic<br>□ Depressive<br>□ Mixed<br>□ Latent	□ Acute<br>□ Stable
I changed the time of day I took my medication				□ **Yes**<br>How did the discussion go?<br>□ **No**<br>Reasons why not	□ Manic<br>□ Depressive<br>□ Mixed<br>□ Latent	□ Acute<br>□ Stable
I stopped my medication earlier than recommended				□ **Yes**<br>How did the discussion go?<br>□ **No**<br>Reasons why not	□ Manic<br>□ Depressive<br>□ Mixed<br>□ Latent	□ Acute<br>□ Stable
I **Never** follow the treatment course I am prescribed				□ **Yes**<br>How did the discussion go?<br>□ **No**<br>Reasons why not	□ Manic<br>□ Depressive<br>□ Mixed<br>□ Latent	□ Acute<br>□ Stable
I **Changed** my behaviour from one pattern to				□ **Yes**<br>How did the discussion go?	□ Manic<br>□ Depressive	□ Acute<br>□ Stable

another, e.g. I used to take more than recommended but stopped doing that				□ **No**<br>Reasons why not	□ Mixed<br><br>□ Latent	
Other – is there anything that you have done that we haven't included in the options here? Please tell us about it!				□ **Yes**<br>How did the discussion go?<br><br>□ **No**<br>Reasons why not	□ Manic<br><br>□ Depressive<br><br>□ Mixed<br><br>□ Latent	□ Acute<br><br>□ Stable
Other – is there anything else that you have done that we haven't included in the options here? Please tell us about it!				□ **Yes**<br>How did the discussion go?<br><br>□ **No**<br>Reasons why not	□ Manic<br><br>□ Depressive<br><br>□ Mixed<br><br>□ Latent	□ Acute<br><br>□ Stable

[service user questionnaire webpage 5]

B.6.. How often do you **deliberately** take your medicines in a way that is different to what has been recommended to you (i.e. in one of the ways described in the previous question)?

☐ Never
☐ Less often than once a month
☐ About once a month
☐ 2-5 times a month
☐ Several times each week
☐ Daily or almost daily

B.7. If you either **always** or **sometimes** take your medicines in the way that is recommended to you, please say briefly what your reasons are for following the recommendations

[service user questionnaire webpage 6]

B.8a. Below are some options for ways in which you might **unintentionally** take your medication in a way that is different to what has been recommended to you by your prescribing doctor. Please tick any that have at some time applied to you.

Options (please tick all that apply)	
**None Apply –** I never unintentionally deviate from recommendations.	☐
I forgot to take my medication / attend my appointment	☐
I lost my prescription	☐
My symptoms prevented me from taking my medication	☐
I was unsure about what my medication recommendations were	☐
I was not able to motivate myself to take my medication	☐
I couldn't get my prescribed medication for practical reasons (such as lack of transport)	☐

[service user questionnaire webpage 7]

B.8b Below are the options you ticked in the previous question for ways in which you might **unintentionally** take your medication in a way that is different to what has been recommended to you by your prescribing doctor. For any that you've ticked, please describe the most recent time you did this and what happened as a result. Tell us whether you discussed this with your doctor and the result of this discussion, or why you didn't discuss it. Finally, if you can, for each option please tell us which phase of your illness you were in at that time.

Options (please tick all that apply)	*Description of most recent time*	*What happened as a result*	*Discussed with doctor? Outcome of discussion or reasons for not discussing*	*Phase of illness **Bipolar Disorder** (Please tick)*	*Phase of illness **Schizophrenia** (Please tick)*
I forgot to take my medication / attend my appointment			□ **Yes**<br>How did the discussion go?<br><br>□ **No**<br>Reasons why not	□ Manic<br>□ Depressive<br>□ Mixed<br>□ Latent	□ Acute<br>□ Stable
I lost my prescription			□ **Yes**<br>How did the discussion go?<br><br>□ **No**<br>Reasons why not	□ Manic<br>□ Depressive<br>□ Mixed<br>□ Latent	□ Acute<br>□ Stable
My symptoms prevented me from taking my medication			□ **Yes**<br>How did the discussion go?<br><br>□ **No**<br>Reasons why not	□ Manic<br>□ Depressive<br>□ Mixed<br>□ Latent	□ Acute<br>□ Stable
I was unsure about what my medication recommendations were			□ **Yes**<br>How did the discussion go?<br><br>□ **No**<br>Reasons why not	□ Manic<br>□ Depressive<br>□ Mixed<br>□ Latent	□ Acute<br>□ Stable
I was not able to motivate myself to take my medication			□ **Yes**<br>How did the discussion go?<br><br>□ **No**<br>Reasons why not	□ Manic<br>□ Depressive<br>□ Mixed<br>□ Latent	□ Acute<br>□ Stable
I couldn't get my prescribed medication for practical reasons (such as lack of transport)			□ **Yes**<br>How did the discussion go?<br><br>□ **No**<br>Reasons why not	□ Manic<br>□ Depressive<br>□ Mixed<br>□ Latent	□ Acute<br>□ Stable

Other - is there anything that has happened to you that we haven't included here? Please tell us about it here:			□ **Yes**<br>How did the discussion go?<br><br>□ **No**<br>Reasons why not	□ Manic<br>□ Depressive<br>□ Mixed<br>□ Latent	□ Acute<br>□ Stable
Other - is there anything else that has happened to you that we haven't included here? Please tell us about it here:			□ **Yes**<br>How did the discussion go?<br><br>□ **No**<br>Reasons why not	□ Manic<br>□ Depressive<br>□ Mixed<br>□ Latent	□ Acute<br>□ Stable

[service user questionnaire webpage 8]

B.9.. How often do you **unintentionally** take your medicines in a way that is different to what has been recommended to you?

☐ Never
☐ Less often than once a month
☐ About once a month
☐ 2-5 times a month
☐ Several times each week
☐ Daily or almost daily

[service user questionnaire webpage 9]

B.10a. Below are some options for different types of support which may help you to get the most out of your treatment. Please tick any types of support that you are **currently** receiving, and tell us how often you receive this type of support. Then tick the types of support you don't currently receive but **would like** to receive. And finally, please tick the three types of support which you feel are or would be the most important in helping you to get the most out of your treatment.

Type of Support	Currently received (tick)	When do you receive this type of support? (tick **all** that apply)	Desired (tick)
Information about the expected benefits of the medication	☐	☐ At the time I get my prescription<br>☐ Every time I see my doctor<br>☐ Only when I ask<br>☐ Once a month<br>☐ Twice a month<br>☐ More than twice a month	☐
Information about the possible side-effects of medication	☐	☐ At the time I get my prescription<br>☐ Every time I see my doctor<br>☐ Only when I ask<br>☐ Once a month<br>☐ Twice a month<br>☐ More than twice a month	☐
Information about other possible treatments (medical or non-medical)	☐	☐ At the time I get my prescription<br>☐ Every time I see my doctor<br>☐ Only when I ask<br>☐ Once a month<br>☐ Twice a month<br>☐ More than twice a month	☐
Information regarding the effect of treatment on likelihood of relapse	☐	☐ At the time I get my prescription<br>☐ Every time I see my doctor<br>☐ Only when I ask<br>☐ Once a month<br>☐ Twice a month<br>☐ More than twice a month	☐
Information about the different styles of administering the therapy (frequency', 'dose', 'route' etc.) and their effect	☐	☐ At the time I get my prescription<br>☐ Every time I see my doctor<br>☐ Only when I ask<br>☐ Once a month<br>☐ Twice a month<br>☐ More than twice a month	☐
Access to a 24 hour anonymous website and/or phone line (independent from your mental health services) where you can ask questions and get information regarding your medication.	☐	☐ At the time I get my prescription<br>☐ Every time I see my doctor<br>☐ Only when I ask<br>☐ Once a month<br>☐ Twice a month<br>☐ More than twice a month	☐
	☐	☐ At the time I get my	☐

Family and peer support in the community		prescription<br>☐ Every time I see my doctor<br>☐ Only when I ask<br>☐ Once a month<br>☐ Twice a month<br>☐ More than twice a month	
Organised peer support groups	☐	☐ At the time I get my prescription<br>☐ Every time I see my doctor<br>☐ Only when I ask<br>☐ Once a month<br>☐ Twice a month<br>☐ More than twice a month	☐
Support for making decisions about treatments	☐	☐ At the time I get my prescription<br>☐ Every time I see my doctor<br>☐ Only when I ask<br>☐ Once a month<br>☐ Twice a month<br>☐ More than twice a month	☐
Frequent and/or sufficiently long meetings with a mental health professional	☐	☐ At the time I get my prescription<br>☐ Every time I see my doctor<br>☐ Only when I ask<br>☐ Once a month<br>☐ Twice a month<br>☐ More than twice a month	☐
Help to reduce the impact of side effects	☐	☐ At the time I get my prescription<br>☐ Every time I see my doctor<br>☐ Only when I ask<br>☐ Once a month<br>☐ Twice a month<br>☐ More than twice a month	☐
Work place support, such as time off to attend appointments	☐	☐ At the time I get my prescription<br>☐ Every time I see my doctor<br>☐ Only when I ask<br>☐ Once a month<br>☐ Twice a month<br>☐ More than twice a month	☐
Practical support, such as childcare during attendance at appointments or transport to appointments	☐	☐ At the time I get my prescription<br>☐ Every time I see my doctor<br>☐ Only when I ask<br>☐ Once a month<br>☐ Twice a month<br>☐ More than twice a month	☐

B.10b.

Type of Support	Tick the five most important types of support for you
Information about the expected benefits of the medication	☐
Information about the possible side-effects of medication	☐
Information about other possible treatments (medical or non-medical)	☐
Information regarding the effect of treatment on likelihood of relapse	☐
Information about the different styles of administering the therapy (frequency', 'dose', 'route' etc.) and their effect	☐
Access to a 24 hour anonymous website and/or phone line (independent from your mental health services) where you can ask questions and get information regarding your medication.	☐
Family and peer support in the community	☐
Organised peer support groups	☐
Support for making decisions about treatments	☐
Frequent and/or sufficiently long meetings with a mental health professional	☐
Help to reduce the impact of side effects	☐
Work place support, such as time off to attend appointments	☐
Practical support, such as childcare during attendance at appointments or transport to appointments	☐

[service user questionnaire webpage 10]

B.11a. Generally speaking, how satisfied are you with your current support? Please put a number between 1 and 5 in the box below, with 1 being not at all satisfied, and 5 being very satisfied.

☐

B.11b. Please tell us briefly the **main** reasons for being satisfied and/or not satisfied with your current support

B.12. Are there any additional types of support you would like to receive that you feel would help you to get the most from your medication?

[service user questionnaire webpage 11]
SECTION C

*****Section C only appears if the six and/or seventh box in q.7 is ticked*****

C. Experience of treatment II: Non-medical treatment
Please answer the questions in the following section in relation to other **non-medical** types of treatment you are receiving for Bipolar Disorder or Schizophrenia, such as talking therapies or support groups, etc.

C.1a. Generally speaking, how satisfied are you with your current treatment? Please put a number between 1 and 5 in the box below, with 1 being not at all satisfied, and 5 being very satisfied.

☐

C.1b. Please tell us the main reasons why you are satisfied and/or not satisfied with your current non-medical treatment.

☐

C.2. When your **non-medical** treatment was decided did your mental health professional discuss your treatment choices with you?

Yes/No

If yes, how involved did you feel in the decision about your treatment? Please put a number between 1 and 5 in the box below, with 1 being not at all involved, and 5 being very involved.

☐

C.3. Generally speaking, when you are following a course of treatment **other than medicine**, how closely would you say you follow the recommendations (such as number of sessions to attend, frequency of sessions, and so on)?

- ☐ I follow the recommendations **exactly**
- ☐ I **mostly** follow the recommendations
- ☐ I do something that is **different** to the recommendations **quite often**
- ☐ I do something that is **different** to the recommendations **most of the time**
- ☐ I **never** follow the recommendations

C.4a. Please tick the statement that best describes how you feel

☐ I would like to follow my treatment recommendations **more** closely than I do
☐ I am satisfied with the way I follow my treatment recommendations and **do not want to change** how closely I follow them
☐ I would like to follow my treatment recommendations **less** closely than I do

C.4b. [If answer to 24a is first or third box:] What help would you need to make the change you want to make?

C.4c. [If answer to 24a is first or third box:] What kind of things have prevented you from following your treatment recommendations in the way that you would like to?

[service user questionnaire webpage 12]

C.5a Below are some options for ways in which you might **deliberately** not follow the recommendations for your treatment course. Please tick any that have at some time applied to you.

Options (tick box for all that apply)	
**None apply -** I **always** follow the treatment course I am recommended	☐
**None Apply** - the only reasons I don't follow the treatment course are unintentional	☐
I missed some of my appointments/sessions for a short period of time	☐
I regularly went to fewer sessions/kept fewer appointments over a long period of time	☐
I accessed more non-medical treatment than my doctor referred me to during a short period of time (i.e. less than a week)	☐
I accessed more non-medical treatment than my doctor referred me to during a long period of time (i.e. a week or more)	☐
I stopped my treatment course earlier than recommended (e.g. stopped going to therapy sessions altogether)	☐
I **Never** follow the treatment course I am recommended	☐
I **Changed** my behaviour from one pattern to another, e.g. went from not attending every session to attending them every time.	☐

[service user questionnaire webpage 13]

Only those options ticked in the previous question appear in the table here.

C.5b Below are the options you ticked in the last question for ways in which you might **deliberately** not follow the recommendations for your treatment course. Please describe the most recent time you did this. What were your reasons, what did you expect to happen and what did actually happen? Please also indicate whether you discussed the change with your mental health professional and if you did, what the result of the discussion was. If you did not discuss the change with your mental health professional, please tell us why not. For each option, if you can, please tell us which phase of your illness you were in at that time.

Options (tick box for all that apply)	*Description of most recent time, including your reasons*	*Expectations*	*What actually happened as a result*	*Discussed with doctor? Outcome of discussion or reasons for not discussing*	*Phase of illness* ***Bipolar Disorder*** *(Please tick)*	*Phase of illness* ***Schizophrenia*** *(Please tick)*
I missed some of my appointments/sessions for a short period of time				□ **Yes** How did the discussion go? □ **No** Reasons why not	□ Manic □ Depressive □ Mixed □ Latent	□ Acute □ Stable
I regularly went to fewer sessions/kept fewer appointments over a long period of time				□ **Yes** How did the discussion go? □ **No** Reasons why not	□ Manic □ Depressive □ Mixed □ Latent	□ Acute □ Stable
I accessed more non-medical treatment than my doctor referred me to during a short period of time (i.e. less than a week)				□ **Yes** How did the discussion go? □ **No** Reasons why not	□ Manic □ Depressive □ Mixed □ Latent	□ Acute □ Stable
I accessed more non-medical treatment than my doctor referred me to during a long period of time (i.e. a week or more)				□ **Yes** How did the discussion go? □ **No** Reasons why not	□ Manic □ Depressive □ Mixed □ Latent	□ Acute □ Stable
I stopped my treatment course earlier than recommended (e.g. stopped going to therapy sessions altogether)				□ **Yes** How did the discussion go? □ **No** Reasons why not	□ Manic □ Depressive □ Mixed □ Latent	□ Acute □ Stable
I **Never** follow the treatment course I am recommended				□ **Yes** How did the discussion go? □ **No** Reasons why not	□ Manic □ Depressive □ Mixed □ Latent	□ Acute □ Stable
I **Changed** my behaviour from one pattern to another, e.g. went from not attending every session to attending them every time.				□ **Yes** How did the discussion go? □ **No** Reasons why not	□ Manic □ Depressive □ Mixed □ Latent	□ Acute □ Stable
Other – is there				□ **Yes**	□ Manic	□ Acute

anything that you have done that we haven't included in the options here? Please tell us about it here:				How did the discussion go?<br><br>□ **No**<br>Reasons why not	□ Depressive<br><br>□ Mixed<br><br>□ Latent	□ Stable
Other – is there anything else that you have done that we haven't included in the options here? Please tell us about it here:				□ **Yes**<br>How did the discussion go?<br><br>□ **No**<br>Reasons why not	□ Manic<br><br>□ Depressive<br><br>□ Mixed<br><br>□ Latent	□ Acute<br><br>□ Stable

[service user questionnaire webpage 14]

C.6. How often do you **deliberately** not follow the recommendations for your treatment course made by a mental health professional (i.e. in one of the ways described in the previous question)?

- ☐ Never
- ☐ Less often than once a month
- ☐ About once a month
- ☐ 2-5 times a month
- ☐ Several times each week
- ☐ Daily or almost daily

C.7. If you either **always** or **sometimes** follow your treatment course in the way that is recommended to you, please say briefly what your reasons are for following the recommendations

[service user questionnaire webpage 15]

C.8a Below are some options for ways in which you might **unintentionally** follow your treatment course in a way that is different to that recommended to you by a mental health professional. Please tick any that have at some time applied to you.

Options (please tick all that apply)	
**None Apply –** I never unintentionally deviate from recommendations.	☐
I forgot to attend my appointment	☐
My symptoms prevented me from accessing the treatment	☐
I was unsure about what my treatment recommendations were	☐
I was not able to motivate myself to follow my treatment course	☐
I was unable to access my treatment for practical reasons (e.g. lack of transport)	☐

[service user questionnaire webpage 16]

C.8b Below are the options you ticked in the previous question for ways in which you might **unintentionally** follow your treatment course in a way that is different to that recommended to you by a mental health professional. For each option, please describe the most recent time you did this and what happened as a result. Tell us whether you discussed this with a mental health professional and the result of this discussion, or why you didn't discuss it. Finally, if you can, for each option please tell us which phase of your illness you were in at that time.

Options (please tick all that apply)	*Description of most recent time*	*What happened as a result*	*Discussed with doctor? Outcome of discussion or reasons for not discussing*	*Phase of illness **Bipolar Disorder***	*Phase of illness **Schizophrenia***
I forgot to attend my appointment			□ **Yes** How did the discussion go?<br>□ **No** Reasons why not	□ Manic<br>□ Depressive<br>□ Mixed<br>□ Latent	□ Acute<br>□ Stable
My symptoms prevented me from accessing the treatment			□ **Yes** How did the discussion go?<br>□ **No** Reasons why not	□ Manic<br>□ Depressive<br>□ Mixed<br>□ Latent	□ Acute<br>□ Stable
I was unsure about what my treatment recommendations were			□ **Yes** How did the discussion go?<br>□ **No** Reasons why not	□ Manic<br>□ Depressive<br>□ Mixed<br>□ Latent	□ Acute<br>□ Stable
I was not able to motivate myself to follow my treatment course			□ **Yes** How did the discussion go?<br>□ **No** Reasons why not	□ Manic<br>□ Depressive<br>□ Mixed<br>□ Latent	□ Acute<br>□ Stable
I was unable to access my treatment for practical reasons (e.g. lack of transport)			□ **Yes** How did the discussion go?<br>□ **No** Reasons why not	□ Manic<br>□ Depressive<br>□ Mixed<br>□ Latent	□ Acute<br>□ Stable
Other - is there anything that has happened to you that we haven't included here? Please tell us about it here:			□ **Yes** How did the discussion go?<br>□ **No** Reasons why not	□ Manic<br>□ Depressive<br>□ Mixed<br>□ Latent	□ Acute<br>□ Stable
Other - is there anything else that has happened to you that we haven't included here? Please tell us about it here:			□ **Yes** How did the discussion go?<br>□ **No** Reasons why not	□ Manic<br>□ Depressive<br>□ Mixed<br>□ Latent	□ Acute<br>□ Stable

[service user questionnaire webpage 17]

C.9. How often do you **unintentionally** change your treatment course in a way that is different to what has been recommended to you?

☐ Never
☐ Less often than once a month
☐ About once a month
☐ 2-5 times a month
☐ Several times each week
☐ Daily or almost daily

[service user questionnaire webpage 18]

C.10. Below are some options for different types of support which may help you to get the most out of your treatment. Please tick any types of support that you are **currently** receiving, and tell us how often you receive this type of support. Then tick the types of support you don't currently receive but **would like** to receive. And finally, please tick the three types of support which you feel are the most important in helping you to get the most out of your treatment.

Type of Support	Currently received (tick)	When do you receive this type of support? (tick **all** that apply)	Desired (tick)	Tick the five most important types of support for you
Information about the expected benefits of the treatment	☐	☐ At the time I get my prescription<br>☐ Every time I see my doctor<br>☐ Only when I ask<br>☐ Once a month<br>☐ Twice a month<br>☐ More than twice a month	☐	☐
Information about the possible side-effects of the treatment	☐	☐ At the time I get my prescription<br>☐ Every time I see my doctor<br>☐ Only when I ask<br>☐ Once a month<br>☐ Twice a month<br>☐ More than twice a month	☐	☐
Information about other possible therapies or treatments	☐	☐ At the time I get my prescription<br>☐ Every time I see my doctor<br>☐ Only when I ask<br>☐ Once a month<br>☐ Twice a month<br>☐ More than twice a month	☐	☐
Information regarding the effect of treatment on likelihood of relapse	☐	☐ At the time I get my prescription<br>☐ Every time I see my doctor<br>☐ Only when I ask<br>☐ Once a month<br>☐ Twice a month<br>☐ More than twice a month	☐	☐
Access to a 24 hour anonymous website and/or phone line (independent from your mental health services) where you can ask questions and get information regarding your medication.	☐	☐ At the time I get my prescription<br>☐ Every time I see my doctor<br>☐ Only when I ask<br>☐ Once a month<br>☐ Twice a month<br>☐ More than twice a month	☐	☐
Family and peer support in the community	☐	☐ At the time I get my prescription<br>☐ Every time I see my doctor<br>☐ Only when I ask<br>☐ Once a month<br>☐ Twice a month<br>☐ More than twice a month	☐	☐
Organised peer support	☐	☐ At the time I get my	☐	☐

groups		prescription<br>☐ Every time I see my doctor<br>☐ Only when I ask<br>☐ Once a month<br>☐ Twice a month<br>☐ More than twice a month		
Support for making decisions about treatments	☐	☐ At the time I get my prescription<br>☐ Every time I see my doctor<br>☐ Only when I ask<br>☐ Once a month<br>☐ Twice a month<br>☐ More than twice a month	☐	☐
Frequent and/or sufficiently long meetings with a mental health professional	☐	☐ At the time I get my prescription<br>☐ Every time I see my doctor<br>☐ Only when I ask<br>☐ Once a month<br>☐ Twice a month<br>☐ More than twice a month	☐	☐
Work place support, such as time off to attend appointments	☐	☐ At the time I get my prescription<br>☐ Every time I see my doctor<br>☐ Only when I ask<br>☐ Once a month<br>☐ Twice a month<br>☐ More than twice a month	☐	☐
Practical support, such as childcare during attendance at appointments or transport to appointments	☐	☐ At the time I get my prescription<br>☐ Every time I see my doctor<br>☐ Only when I ask<br>☐ Once a month<br>☐ Twice a month<br>☐ More than twice a month	☐	☐

[service user questionnaire webpage 19]

C.11a Generally speaking, how satisfied are you with your current support? Please put a number between 1 and 5 in the box below, with 1 being not at all satisfied, and 5 being very satisfied.

C.11b. Please tell us briefly the **main** reasons for being satisfied and/or not satisfied with your current support

C.12. Are there any additional types of support you would like to receive that you feel would help you to get the most from your treatment?

[service user questionnaire webpage 20]

D. Please answer the following question in relation to all treatments (medical and non-medical) which you are receiving.

D.1. Tell us about **any** ways that those around you (including friends / family / mental health professionals) could better help you to get the most from your treatment.

Thank you!
